# Predicting OptimaL cAncer RehabIlitation and Supportive care (POLARIS): rationale and design for meta-analyses of individual patient data of randomized controlled trials that evaluate the effect of physical activity and psychosocial interventions on health-related quality of life in cancer survivors

**DOI:** 10.1186/2046-4053-2-75

**Published:** 2013-09-13

**Authors:** Laurien M Buffart, Joeri Kalter, Mai JM Chinapaw, Martijn W Heymans, Neil K Aaronson, Kerry S Courneya, Paul B Jacobsen, Robert U Newton, Irma M Verdonck-de Leeuw, Johannes Brug

**Affiliations:** 1 Department of Epidemiology and Biostatistics, and the EMGO Institute for Health and Care Research, VU University Medical Center, Van der Boechorststraat 7, 1081 BT, Amsterdam, The Netherlands; 2 Department of Public and Occupational Health and the EMGO Institute for Health and Care Research, VU University Medical Center, Van der Boechorststraat 7, 1081 BT, Amsterdam, The Netherlands; 3 The Netherlands Cancer Institute, Plesmanlaan 121, 1066 CX, Amsterdam, The Netherlands; 4 Faculty of Physical Education and Recreation, University of Alberta, E-488 Van Vliet Center, Edmonton, Alberta T6G 2H9, Canada; 5 Moffitt Cancer Center and Research Institute, 12902 Magnolia Drive, MRC-PSY, Tampa, Florida 33612, USA; 6 Edith Cowan University Health and Wellness Institute, Edith Cowan University, 270 Joondalup Drive, Joondalup, WA 6027, Australia; 7 Department of Clinical Psychology, VU University, Van der Boechorststraat 1, 1081 BT, Amsterdam, The Netherlands; 8 Department of Otolaryngology - Head & Neck Surgery, VU University Medical Center, De Boelelaan 1117, 1081 HV, Amsterdam, The Netherlands

**Keywords:** Physical activity, Exercise, Neoplasms, Psychotherapy, Health-related quality of life, Rehabilitation, Individual patient data meta-analysis

## Abstract

**Background:**

Effective interventions to improve quality of life of cancer survivors are essential. Numerous randomized controlled trials have evaluated the effects of physical activity or psychosocial interventions on health-related quality of life of cancer survivors, with generally small sample sizes and modest effects. Better targeted interventions may result in larger effects. To realize such targeted interventions, we must determine which interventions that are presently available work for which patients, and what the underlying mechanisms are (that is, the moderators and mediators of physical activity and psychosocial interventions). Individual patient data meta-analysis has been described as the ‘gold standard’ of systematic review methodology. Instead of extracting aggregate data from study reports or from authors, the original research data are sought directly from the investigators. Individual patient data meta-analyses allow for adequate statistical analysis of intervention effects and moderators of such effects.

Here, we report the rationale and design of the Predicting OptimaL cAncer RehabIlitation and Supportive care (POLARIS) Consortium. The primary aim of POLARIS is 1) to conduct meta-analyses based on individual patient data to evaluate the effect of physical activity and psychosocial interventions on the health-related quality of life of cancer survivors; 2) to identify important demographic, clinical, personal, or intervention-related moderators of the effect; and 3) to build and validate clinical prediction models identifying the most relevant predictors of intervention success.

**Methods/Design:**

We will invite investigators of randomized controlled trials that evaluate the effects of physical activity and/or psychosocial interventions on health-related quality of life compared with a wait-list, usual care or attention control group among adult cancer survivors to join the POLARIS consortium and share their data for use in pooled analyses that will address the proposed aims. We are in the process of identifying eligible randomized controlled trials through literature searches in four databases. To date, we have identified 132 eligible and unique trials.

**Discussion:**

The POLARIS consortium will conduct the first individual patient data meta-analyses in order to generate evidence essential to targeting physical activity and psychosocial programs to the individual survivor’s characteristics, capabilities, and preferences.

**Registration:**

PROSPERO: International prospective register of systematic reviews, CRD42013003805

## Background

Worldwide, it has been estimated that there were about 12.7 million cancer cases and 7.6 million cancer deaths in 2008 [1]. Due to advances in early detection and treatment, survival after cancer diagnosis has improved substantially. Nevertheless, for most patients, cancer survivorship (that is, from the time of diagnosis [2]) is associated with significant adverse physical and psychosocial problems. These include fatigue, pain, increased risk of anxiety and depression, reduced physical fitness and physical function [3,4], and impaired health-related quality of life (HRQoL) [5,6]. The term HRQoL denotes a range of health outcomes and effects, including physical, mental and social functioning, symptom burden and perceived health status [7,8].

A range of physical activity and psychosocial interventions targeting HRQoL outcomes in cancer survivors have been developed and evaluated. Many of these interventions have been studied in the context of a randomized controlled trial (RCT). In general, meta-analyses of these RCTs have yielded significant, positive results, although the mean effect sizes tend to be small to moderate [9-12].

One possible explanation for the lack of larger effect sizes is that these interventions are typically offered to a heterogeneous group of cancer survivors and are not sufficiently targeted to specific patients. Also, the use of different HRQoL definitions and assessment tools undoubtedly contributes to the relatively wide range of findings regarding the strength of intervention effects. Finally, determinants of HRQoL may vary between individuals and change over time. Thus, similar to developments in personalized primary cancer therapy, physical activity and psychosocial interventions should be optimally targeted to the individual’s characteristics, health state, needs, preferences, capabilities and opportunities.

To be able to shift from a ‘one-size-fits-all’ approach to more personalized physical activity and psychosocial interventions, it is essential to know *which* existing programs work, for whom, and under what circumstances (that is, to identify important moderators of intervention effects). Moderators identify which (subgroups of) patients are most responsive to the intervention, and which are not responsive, providing valuable information for decision-making [13]. The few published studies of potential moderators of the effects of physical activity and psychosocial interventions have suggested that demographic, clinical and personal factors such as age, marital status, disease stage, type of treatment, and baseline functioning may help to understand differences in responses to physical activity and psychosocial interventions [14-18]. However, most of these earlier reports were based on single studies that were not designed or powered to analyze moderating effects and conduct subsequent stratified analyses.

To further improve the effectiveness and efficiency of physical activity and psychosocial interventions, it is also important to identify and subsequently target critical intervention components (that is, mediators of intervention effect). Mediators are causal links between the intervention and the outcome and identify how an intervention might achieve its effects. For example, previous studies have shown that fatigue and psychological distress may mediate the association between physical activity and HRQoL [19,20]. However, such studies are scarce.

An individual patient data (IPD) meta-analysis has been suggested as the preferred method to identify moderators of intervention effects [21]. In contrast to meta-regression analyses of aggregated data used in study-level meta-analyses, an IPD meta-analysis allows for testing of interactions to evaluate whether patient and setting characteristics are related significantly to treatment effects [21]. Other key benefits of an IPD meta-analysis include the larger number of data points, facilitating more powerful statistical conclusions based on careful evaluation of modeling assumptions and accounting for missing data at the individual patient level, the ability to standardize analytical techniques, inclusion criteria and outcome definitions across studies, the possibility of identifying relevant subgroups, and the ability to develop and test new and existing prediction models [22-24].

In this paper, we describe the protocol of the Predicting OptimaL cAncer RehabIlitation and Supportive care (POLARIS) project. The primary objectives of the POLARIS project are (1) to conduct IPD meta-analyses to evaluate the effects of physical activity and psychosocial interventions on the HRQoL of cancer survivors; (2) to identify those demographic, clinical and personal characteristics, and intervention types and circumstances that moderate the effects of physical activity and psychosocial interventions; and (3) to build and validate clinical prediction models that identify the most relevant predictors of intervention success (that is, improvement in HRQoL). The secondary aim of the project is to explore which variables mediate the effect of physical activity and psychosocial interventions on HRQoL.

To our knowledge, this is the first IPD meta-analysis conducted on the effects of physical activity and psychosocial interventions on HRQoL of cancer survivors. For the POLARIS project, we have established a consortium that will be expanded to include as many investigators as possible who have conducted RCTs evaluating the effects of physical activity and/or psychosocial interventions on HRQoL.

## Methods/Design

### Inclusion and exclusion criteria

For POLARIS, we will include RCTs conducted among adult cancer survivors where the effects of physical activity and/or psychosocial interventions on HRQoL are evaluated in comparison to a wait-list, usual care or attention control group (Table 1). In addition, the RCTs should have approval of a Medical Ethics Committee as well as signed informed consent of each participant. Psychosocial inter-ventions will be included if they fit into the first four categories of the framework proposed by Cunningham [25]. This framework classifies psychosocial interventions into five categories: 1) patient education; 2) social support; 3) coping skills training; 4) psychotherapy; and 5) spiritual/existential therapy. Although we acknowledge the importance, we will initially exclude studies focusing on spiritual or existential therapy, including meditation and mindfulness, in order to reduce the heterogeneity among the interventions to be included. We also excluded studies focusing on yoga, pain management, diet or multimodal lifestyle interventions (for example, physical activity and diet combined).

**Table 1:** Study inclusion criteria

1.	Study design	Randomized controlled trial	
2.	Patients	Adult (≥ 18 years) cancer survivors	
3.	Intervention	Physical activity or psychosocial intervention	
		*Physical activity intervention*	*Psychosocial intervention*^*1*^
		Physical activity advise or education	Providing information/counseling
		Aerobic exercise	Support groups
		Resistance exercise	Coping skills training
		Combination	Psychotherapy
4.	Control group	Wait-list, usual care or attention control	
5.	Outcome	Health-related quality of life included as primary or secondary outcome measure	

^1^According to the Framework proposed by Cunningham [25].

### Identification and selection of studies

We used several strategies to identify eligible studies, including literature searches and personal communication with experts in the field, collaborators and colleagues. Electronic databases of PubMed, EMBASE, PsycINFO, and CINAHL were searched, without language restrictions, to obtain an overview of studies published. Because of language barriers, for the time being we have only included articles published in English, German or Dutch. We used medical subject heading (MESH) and text words related to cancer, physical activity, exercise (that is, form of physical activity that is planned - structured and repetitive - and aims to improve fitness, performance or health [26]), psychosocial therapy, (health-related) quality of life, randomized controlled trials and adult. Detailed search strategies of all databases are available on request [See Additional file 1 for the strategy in PubMed]. We identified additional records by examining other sources (that is, systematic reviews, meta-analyses, personal communication with experts in the field, collaborators and colleagues) until no further studies were found.

**Figure 1:**
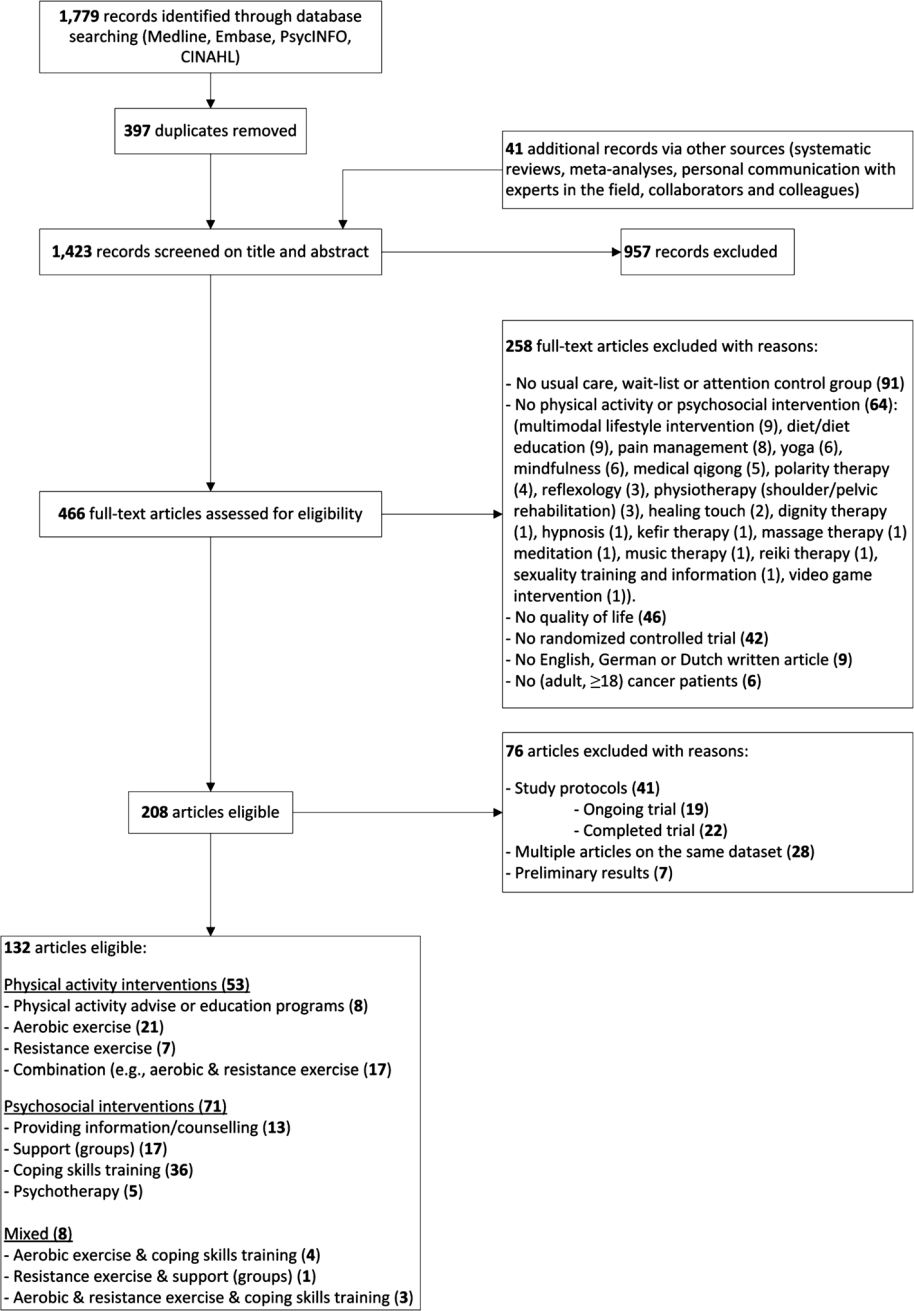
Flow chart of study selection.

To date, based on the search through September 2012, we have identified a total of 1,779 records through database searching, and an additional 41 records through other sources (Figure 1). After removing duplicates, we screened 1,423 records on title and abstract, of which 957 were out of scope. We assessed full text articles of 466 records for eligibility, of which 208 met the inclusion criteria. We excluded 76 of these articles because they were descriptions of a study protocol, or were multiple publications from the same trial. Finally, 132 unique RCTs met our inclusion criteria (Table 1). We will invite the principal investigators of all 132 studies to participate in the POLARIS consortium. This will involve sharing their trial data and participating in analyses and manuscript preparation (see below).

### Core data set and variables

The main outcome measures are overall HRQoL and specific HRQoL domains (for example, physical, psychological, functional, and social well-being) measured such multidimensional questionnaires as the European Organization for Research and Treatment of Cancer Quality of Life Questionnaire - Core 30 (EORTC QLQ-C30) [27], the Short Form-36 Item Health Survey(SF-36) [28] and its abbreviated version, the SF-12 [29], the Functional Assessment of Chronic Illness Therapy (FACIT) [30], the Functional Assessment of Cancer Therapy (FACT) [31], and the EuroQol 5D (EQ5D) [32]. Other patient-related outcomes of interest and baseline characteristics include physical activity (measured by self-report and/or objective assessment instruments) and physical fitness (for example, peak oxygen uptake (VO_2_)), body composition, symptoms (for example, fatigue) and psychosocial variables including anxiety, depression, distress, mood, self-esteem, sleep quality and social support (Table 2). No outcome measure will be excluded *a priori*.

**Table 2:** Overview primary, secondary outcome and independent variables

**Primary outcome measures**	**Assessment instrument**
Health-related quality of life	For example, EORTC QLQ C30, FACIT, FACT, SF-36, SF-12, EQ5D.
**Secondary outcome measures and independent variables**	**Variable name**
Psychosocial factors	Fatigue, depression, anxiety, mood state, stress/distress, self-esteem, anger, sleep quality, social support.
Physical activity and fitness	Functional performance (for example, 6 min walk test), muscle strength, aerobic fitness (for example, peak VO_2_), physical activity (objectively or by self-report).
Body composition	Height, weight, body mass index, fat mass, lean body mass, thickness of skin folds, body fat (in percentages), arm circumference, waist circumference, hip circumference, waist-hip ratio, bone mineral density.
Baseline characteristics	Patient identifier, center identifier, date of diagnosis, time since diagnosis, date of randomization, and timing of intervention (pre/during/post intervention or mixed timing).
Demographic variables	Age, gender, family income, employment status, level of education, marital status, ethnicity/race, smoking, alcohol use, menopausal status, performance status (for example, Karnofsky Performance Scale).
Clinical characteristics	Cancer diagnosis (for example, breast cancer), cancer staging and grading, TNM Classification of Malignant Tumors, oncologic history, recurrence of cancer, co-morbidities, treatment of co-morbidities, cancer-related pain, medication use, type of medication, type of treatment (for example, chemo/radio/hormone therapy), number of cycles, time since treatment, currently under treatment, complications during treatment, other treatments used (for example, immunotherapy, stem cell transplantation).
Psychosocial intervention characteristics	Method of delivery (for example, telephone support, face-to-face), intervention type (for example, education, cognitive behavioral therapy, psychotherapeutic), intervention format (for example, group, individual, couples, web-based), total number of sessions of the intervention, number of care providers involved in the intervention, profession of care providers involved in the intervention, training given to the care providers involved in the intervention, compliance.
Physical activity intervention characteristics	Intervention duration, exercise mode (for example, resistance, endurance), exercise intensity, exercise frequency, exercise session duration, exercise supervision, compliance.

EORTC QLQ C30, European Organization for Research and Treatment of Cancer Quality of Life Questionnaire Core 30; EQ5D, EuroQoL 5D; FACIT, Functional Assessment of Chronic Illness Therapy; FACT, Functional Assessment of Cancer Therapy; peak VO_2,_ peak oxygen consumption; SF-36, Short Form-36; SF-12, Short Form-12; TNM, tumor node metastasis.

Relevant baseline characteristics to be included in the POLARIS database include the patient and center identifier, important demographic and clinical variables, as well as intervention characteristics (Table 2).

### Establishing the collaborative group

The POLARIS Steering Committee will send a letter of invitation to join the POLARIS consortium to the principal investigator of each study that is eligible for the POLARIS database. This invitation contains a short introduction to POLARIS, including the aim and inclusion criteria, and a short description of the POLARIS policy and procedures. Reminders will be sent to principal investigators who do not respond to the first letter of invitation, and telephone contact will be sought. If necessary, another (principle) investigator involved in the project will be contacted. If, and when, the principal investigators express interest in joining the consortium and sharing their data, they will be asked to provide more trial information and to describe which data they are willing to share with the POLARIS database. Further, the full POLARIS policy and a data sharing agreement form will be sent to the principal investigator. Reasons for refusal will be recorded. After receiving the signed data sharing agreement form, a data transfer protocol will be sent with a suggested data-coding scheme allowing flexibility in the format to ensure convenience to all collaborators. Alternatively, if data management support is needed, the dataset may be transferred with the original coding scheme.

### Data acquisition, collection and checking

We will ask study collaborators to supply raw data as outlined by the data request form. The data can be transferred in any electronic format (for example, SPSS, SAS, and STATA). Data will be transferred using a password-protected encryption (for example, AxCrypt). Once the original data file is received from the principal investigator, it will be transferred to SPSS (IBM SPSS Statistics for Windows, Version 20.0. Armonk, NY, USA) and the original data will be archived for backup purposes.

Before transferring the data to the POLARIS database, the data sets must be anonymized by the original investigators (that is, have all directly identifiable material, including name, address, postal code or medical record number removed). A unique patient identification number should be provided to facilitate communication and data queries.

We will examine the original data for completeness and consistency using the following protocol: summary statistics for all variables will be sent back to collaborators to verify categories, units of measurements, and comparing baseline characteristics with previous publications. In addition, we will verify consistency of data within individuals, highlight potential outliers and identify missing data. Any data queries will be discussed and resolved directly with the responsible collaborating principal investigator.

### Harmonization

**Figure 2:**
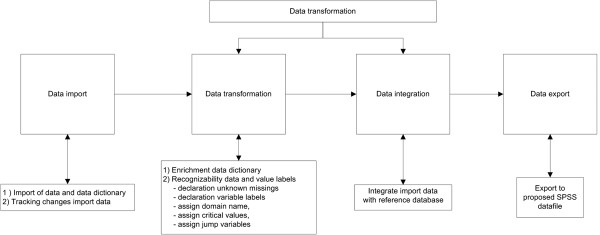
Data harmonization process.

To harmonize variables, we will collect information from all studies and follow a conversion procedure consisting of four steps: (1) importation of data into the data warehouse; (2) preparation for transformation of original studies, including variable checking; (3) transformation of the data labels of the original studies into the POLARIS coding scheme and integration into the data warehouse; and (4) export of specific variables into a SPSS data file for the proposed statistical analyses. POLARIS data management processes from the original data sets from collaborating principal investigators to the formation of the POLARIS database is described in more detail in Figure 2.

### Data confidentiality

Data made available for the POLARIS database will remain the property of the investigators supplying the data. Any data supplied will be held securely at the EMGO Institute for Health and Care Research and will be treated as confidential. All data included in the POLARIS project will be anonymized by the principal investigators prior to data transfer to the POLARIS center (if this has not already been done). Only RCTs that had ethical committee approval will be included in the POLARIS database.

### Statistical analysis

To conduct the statistical analyses, we will pool individual patient data from RCTs contained in the POLARIS database. We will conduct one-stage IPD meta-analyses to evaluate the effect of physical activity and psychosocial interventions on HRQoL compared with wait-list, usual care or attention control group. Clustering effects from study to study will be taken into account by using multilevel regression analyses with a two-level hierarchical structure: the patients within each trial as level 1 and the trial as level 2.

#### Moderators

To test for moderating effects, we will use moderated multiple regression analyses (MMR) [33]. MMR is an extension of a multiple regression equation that includes an interaction term providing information regarding a potential moderating effect. The selection of moderators will be based on a specific rationale (that is, a theory or evidence-based model) of why the intervention may be more effective for some subgroups than for others. We will examine interactions between the intervention and potential categorical moderators (that is, demographic, clinical and personal factors plus treatment such as age, marital status, disease stage, type of treatment (for example, chemotherapy) and baseline functioning). The regression coefficient of the interaction term provides information on whether the effect of the intervention on the outcome differs across different moderator categories. Before conducting MMR, we will check the homogeneity of (within-group) error variance, that is, whether the error variance for one moderator group is equal to the error variance in the other moderator group(s) [33]. We will do this by examining whether the residual variance is constant across the moderator categories.

#### Predictors

For each type of intervention, we will build prediction models identifying predictors of intervention success (that is, improvement in HRQoL), using multivariable backward logistic regression analyses on pooled data [34,35]. We will explore the need to account for trial variability in these models. The variables with the highest *P* values will be removed one by one, based on the Wald test, until all remaining variables have a significant pre-determined *P* value. Potential predictors include demographic, clinical and personal and treatment characteristics at baseline. Relevant moderators identified will also be taken into account when building the prediction models. In addition, we will build a clinical prediction model to select the most successful intervention to improve HRQoL for (subgroups of) patients. The predictors included in the model will be checked for interactions with treatment by introducing interaction terms into the model, and evaluating their contribution to the model. We will calculate the probabilities of success for the different categories of the predictors interacting with treatment [36]. Finally, we will try to translate the clinical prediction model into a clinical decision rule that may assist patients and clinicians in making the most objective, evidence-based and well-considered choice for optimal physical activity or psychosocial interventions to improve HRQoL. This model may guide treatment choice and may predict which patient will benefit most from a specific treatment.

The performance of the prediction models will be evaluated using the Hosmer-Lemeshow goodness-of-fit test, and the discriminative ability of the regression model using the area under the receiver operating characteristics (ROC) curve and its 95% confidence interval. Internal validation of the model will be determined by a bootstrapping procedure with 200 replications. In each replication, a random sample from the original dataset is drawn with replacement. We will multiply the regression coefficients by the shrinkage factor derived from the bootstrapping procedures to quantify the amount of optimism and to correct for over-fitting if necessary.

#### Mediators

**Figure 3:**
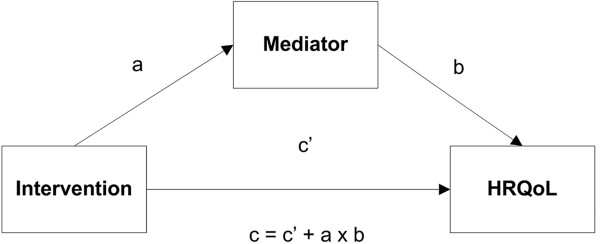
Mediation analysis.

Potential mediators of the intervention effect on HRQoL will be explored according to the product‒of‒coefficients test described by MacKinnon (Figure 3) [37]. The selection of mediators will be based on the theoretical framework of the included studies. First, we will estimate the total intervention effect on the outcome (path c). Second, we will estimate the intervention effect of the hypothesized mediator (path a). Third, we will estimate the association between the mediator and outcome, adjusted for the intervention effect (path b). The final regression model provides estimates for the b‒value and for the direct association (c’‒path). The product of coefficients (a × b) provides an estimate of the relative strength of the mediation effect. The proportion mediated will be estimated by dividing the mediation effect (a × b) by the total direct effect (c = c’ + a × b). Subsequently, a bootstrapping method (with n = 5,000 bootstrap resamples) will be used to calculate the bias corrected confidence intervals around the mediated and direct effects using the SPSS macro suggested by Preacher and Hayes [38]. In case of multiple mediators, path models and structural equation models will be constructed [37].

### Project management

A Steering Committee (that is, LMB, JK, IMVdL, JB) has been established and is responsible for the coordination of the POLARIS project, advised by an international advisory board consisting of experts in this research field (that is, NKA, KSC, PBJ, RUN). Project coordination and statistical analyses will be conducted at the EMGO Institute for Health and Care Research and the Department of Epidemiology and Biostatistics of VU University Medical Center, Amsterdam. Collaborating investigators are welcome to propose additional research projects, to develop analysis protocols and to spend time at the coordinating center conducting data analysis. The steering committee will check for potential overlap with other proposals, and subsequently, all collaborators will be contacted to ask permission for the use of their data for the proposed analysis. Collaborators may decline participation on a study-by-study basis, and have the right to withdraw their data for future analyses.

### Publication policy

The results of the specific meta-analyses will be presented to and discussed with all collaborators during a collaborators meeting. Subsequently, the results will be published in scientific peer-reviewed journals. The primary publications will be in the name of the writing committee as well as the collaborative group. The writing committee for these primary publications will consist of the research staff working in the analysis center and those collaborators who have expressed interest in that particular analysis. All co-authors need to comply with the criteria of the Vancouver Protocol for co-authorship. The POLARIS consortium will be listed as a group author, and all participating studies and investigators contributing to this project will be listed at the end of each publication.

## Discussion

The POLARIS consortium will conduct the first IPD meta-analyses based on individual patient data, with the goal of more effectively targeting physical activity or psychosocial programs to cancer survivors. Furthermore, insight into the moderators explaining which physical activity or psychosocial intervention can improve HRQoL for whom and under what circumstances is an essential step towards personalized care for cancer survivors. IPD meta-analysis allows for testing of interactions to evaluate whether patient and setting characteristics are statistically significantly related to treatment effects. Further, it may allow us to build a clinical decision rule supporting evidence-based decision making about which intervention would be most effective for a given outcome and a given patient group. This can be an essential step to improve care and optimize the patient’s HRQoL in an efficient and evidence-based way. It may also help to identify subgroups of patients for which effective interventions are not yet available and thus need to be developed and evaluated.

Despite the strong study design allowing sophisticated statistical analyses, an IPD meta-analysis is at risk for ‘retrieval bias’ if not all investigators of relevant studies are willing or able to participate. However, estimated effect sizes may still be valid because it is unlikely that non-participation is associated with effect size.

In summary, the POLARIS consortium will start to carry out a series of IPD meta-analyses evaluating the effectiveness of physical activity and psychosocial interventions on the HRQoL of cancer survivors in order to identify relevant moderators of intervention effects, and will try to build a clinical prediction rule that may support evidence-based decision making about which interventions are most likely to be effective at the individual patient level.

## Abbreviations

EORTC QLQ C30: European organization for research and treatment of cancer quality of life questionnaire core 30; EQ5D: Euroqol 5D; FACIT: Functional assessment of chronic illness therapy; FACT: Functional assessment of cancer therapy; HRQoL: Health-related quality of life; IPD: Individual patient data; MESH: Medical subject heading; MMR: Moderated multiple regression analyses; POLARIS: Predicting optimal cancer rehabilitation and supportive care; RCT: Randomized controlled trial; ROC: Receiver operating characteristics; SF: Short form.

## Competing interests

The authors declare that they have no competing interests.

## Authors’ contributions

LMB and JK drafted the manuscript. MJMC, MWH, NKA, KSC, PBJ, RUN, IMVdL and JB critically reviewed the manuscript. All authors read and approved the final manuscript.

## Supplementary Material

Additional file 1 Search strategy of PubMed (MEDLINE). Click here for file
