# Supplementary material for: Predicting OptimaL cAncer RehabIlitation and Supportive care (POLARIS): rationale and design for meta-analyses of individual patient data of randomized controlled trials that evaluate the effect of physical activity and psychosocial interventions on health-related quality of life in cancer survivors
Source: Syst Rev. 2013 Sep 13;2:75. doi: 10.1186/2046-4053-2-75 (PMC3848838; doi:10.1186/2046-4053-2-75)
Supplement: Additional file 1 — Search strategy of PubMed (MEDLINE). [file 2046-4053-2-75-S1.pdf]

Search neoplasms - “neoplasms”[Mesh] OR metastas*[tiab] OR neoplas* [tiab] OR tumour*[tiab] OR cancer*[tiab] OR tumor[tiab] OR tumora*[tiab] OR tumorb*[tiab] OR tumorc*[tiab] OR tumord*[tiab] OR tumore*[tiab] OR tumorf*[tiab] OR tumorg*[tiab] OR tumorh*[tiab] OR tumori*[tiab] OR tumork*[tiab] OR tumorl*[tiab] OR tumorm*[tiab] OR tumorn*[tiab] OR tumoro*[tiab] OR tumorp*[tiab] OR tumorr*[tiab] OR tumors*[tiab] OR tumort*[tiab] OR tumoru*[tiab] OR tumorv*[tiab] OR tumorw*[tiab] OR tumorx*[tiab] OR tumory*[tiab] OR tumorz*[tiab] OR tumor’*[tiab] OR tumor1[tiab]

Search exercise therapy - “exercise therapy”[Mesh] OR exercise therap*[tiab] OR “physical education and training” [Mesh] “physical education”[tiab] OR “physical Fitness”[Mesh] OR “physical fitness”[tiab] OR “walking”[Mesh] OR “walking”[tiab] OR kinesiotherap*[tiab] OR “walking training”[tiab] OR “exercise treatment”[tiab] OR therapeutic exercis*[tiab] OR “calisthenics”[tiab] OR gymnastic*[tiab]

Search psychosocial therapy - “Social Support”[Mesh] OR “Behavior Therapy”[Mesh] OR “cognitive therapy”[Mesh] OR “Mind-body therapies”[Mesh] OR “relaxation therapy”[Mesh] OR “counseling”[Mesh] OR “biofeedback, psychology”[Mesh] OR “guideline adherence”[Mesh] OR “patient compliance”[Mesh] OR “patient education as topic”[Mesh] OR “Health promotion”[Mesh] OR “Health education”[Mesh] OR “health behavior”[Mesh] OR “Reinforcement (Psychology)”[Mesh] OR “social support”[tiab] OR “Behavior therapy”[tiab] OR “cognitive therapy”[tiab] OR “Mind-body therapies”[tiab] OR counselor* [tiab] OR “psychology biofeedback”[tiab] OR “guideline adherence”[tiab] OR “patient compliance”[tiab] OR “patient education as topic”[tiab] OR “Health promotion”[tiab] OR “Health education”[tiab] OR “health behavior”[tiab] OR “Reinforcement (Psychology)”[tiab] OR alternative therap*[tiab] OR “Psychophysiology”[tiab] OR “behavior training”[tiab] OR “behavior treatment”[tiab] OR “desensitization”[tiab] OR “CBT”[tiab] OR cognitive behavior therap*[tiab] OR cognitive behavior treatment*[tiab] OR cognitive behavioral therap*[tiab] OR cognitive behavioral treatment*[tiab] OR cognitive behaviour therap*[tiab] OR cognitive behaviour treatment*[tiab] OR cognitive behavioural therap*[tiab] OR cognitive behavioural treatment*[tiab] OR “anthroposophy”[tiab] OR “complementary medicine”[tiab] OR complementary therap*[tiab] OR mind-body relation*[tiab] OR mind-body therap*[tiab] OR mind body techniq*[tiab] OR mind body therap*[tiab] OR “naturopathy orthomolecular medicine”[tiab] OR polarity thera*[tiab] OR reflexotherap*[tiab] OR spiritual therap*[tiab] OR “mind-body and relaxation techniques”[tiab] relaxation therap*[tiab] OR client centered therap*[tiab] OR nondirective therap*[tiab] OR “biofeedback (psychology)”[tiab] OR psychoneuroimmunolog*[tiab] OR psychophysiologic respons*[tiab] OR “patient adherence”[tiab] OR “treatment compliance”[tiab] OR health behav*[tiab] OR health promoting behav*[tiab] OR health related behav*[tiab] OR “conditioning”[tiab] OR “differential reinforcement”[tiab] OR “knowledge of results (psychology) ”[tiab]

Search quality of life - “quality of life”[Mesh] OR “quality of life”[tiab] OR “QoL”[tiab] OR “Health related quality of life”[tiab] OR “HRQL”[tiab] OR “life quality”[tiab]

Search RCT - “randomized controlled trial”[pt] OR “controlled clinical trial”[pt] OR “randomized controlled trials”[mh] OR “random allocation” [mh] OR “double-blind method” [mh] OR “single-blind method” [mh] OR “clinical trial” [pt] OR “clinical trials” [mh] OR "clinical trial" [tw] OR ((singl* [tw] OR doubl* [tw] OR trebl* [tw] OR tripl* [tw]) AND (mask* [tw] OR blind* [tw])) OR "latin square" [tw] OR placebos [mh] OR placebo* [tw] OR random* [tw] OR research design [mh:noexp] OR comparative study [pt] OR evaluation studies [pt] OR follow-up studies [mh] OR prospective studies [mh] OR cross-over studies [mh] OR control[tw] OR controll*[tw] OR prospectiv* [tw] OR volunteer* [tw]) NOT (animal [mh] NOT human [mh])

Search Adult - (("Adolescent"[Mesh] OR "Child"[Mesh] OR "Infant"[Mesh] OR adolescen*[tiab] OR child*[tiab] OR schoolchild*[tiab] OR infant*[tiab] OR girl*[tiab] OR boy*[tiab] OR teen[tiab] OR teens[tiab] OR teenager*[tiab] OR youth*[tiab] OR pediatr*[tiab] OR paediatr*[tiab] OR puber*[tiab]) NOT ("Adult"[Mesh] OR adult*[tiab] OR man[tiab] OR men[tiab] OR woman[tiab] OR women[tiab]))
